# Characterization of lncRNAs contributing to drug resistance in epithelial ovarian cancer

**DOI:** 10.1007/s12032-025-02628-1

**Published:** 2025-02-24

**Authors:** Ehteram Khademi Siahestalkhi, Aydin Demiray, Arzu Yaren, Atike Gökçen Demiray, Seçil Tan, Hakan Akça

**Affiliations:** 1 Department of Medical Genetics, Faculty of Medicine, Pamukkale University, Denizli, Turkey; 2 Department of Internal Medicine, The Affiliated Hospital of Pamukkale University, Denizli, Turkey

**Keywords:** Biomarker, Carboplatin, Drug resistance, Epithelial ovarian cancer, Long noncoding RNA, Paclitaxel

## Abstract

Epithelial ovarian cancer (EOC) is the second leading cause of death among women with gynecological cancers, particularly in high-income countries. Despite significant advancements in molecular oncology and an initially positive response to primary chemotherapy, the development of drug resistance remains a major challenge in the effective management of EOC. Consequently, there is an urgent need for innovative biological markers that can enable early diagnosis and provide more accurate predictions of recurrence risk in ovarian cancer patients. This study investigated the expression profiles of seven specific long noncoding RNAs (lncRNAs)—SNHG7, TUG1, XIST1, PRLB, TLR8-AS1, ZFAS1, and PVT1—associated with epithelial ovarian cancer and their relationship with drug resistance. To achieve this, drug-resistant subtypes of aggressive EOC cell lines, including carboplatin/paclitaxel-resistant OVCAR3 and SKOV3 lines, were developed. The expression profiles of the selected lncRNAs were quantitatively analyzed using RT-qPCR across various ovarian cancer cell lines and in serum samples from 25 patients before chemotherapy, six months after treatment, and 23 healthy controls. The findings revealed that the target lncRNAs were significantly upregulated under drug-resistant conditions and in post-chemotherapy serum samples, suggesting their involvement in a complex regulatory network. These results highlight the critical roles of lncRNAs in the progression and treatment response of EOC, positioning them as potential therapeutic targets and biomarkers for early diagnosis and treatment stratification. Identifying reliable lncRNA biomarkers could enable the early detection of patients at risk for developing drug resistance, thereby facilitating personalized treatment strategies to improve patient outcomes and survival rates.

**Supplementary Information:**

The online version contains supplementary material available at 10.1007/s12032-025-02628-1.

## Introduction

Epithelial ovarian cancer (EOC) is a highly heterogeneous and aggressive malignancy, predominantly originating from the epithelium of the fallopian tubes [1]. Serous tubal intraepithelial carcinoma (STIC) lesions are now widely recognized as precursors to high-grade serous ovarian cancer (HGSOC), the most common and lethal subtype of EOC [2]. This paradigm underscores the fallopian tube epithelium’s critical role in EOC’s pathogenesis [3]. Early mutational events, such as TP53 mutations, initiate clonal expansion within the fimbrial epithelium, while concurrent defects in DNA repair pathways—most notably involving BRCA1/2 and RAD51C/D—contribute to genomic instability, a hallmark of tumor progression [4]. Recent investigations have identified additional non-canonical DNA repair mutations, such as those in PALB2 and FANCM, which represent potential therapeutic targets beyond conventional homologous recombination deficiency (HRD) testing [5, 6]. Collectively, these molecular alterations, along with intricate interactions between tumor cells and the microenvironment, drive the aggressive clinical behavior and poor prognosis associated with EOC [7].

Globally, EOC constitutes a significant public health burden, with over 324,000 new cases and 206,000 deaths reported in 2022 [8]. Despite advancements in diagnostic modalities and therapeutic interventions, the 5-year survival rate for EOC remains approximately 47%, largely dependent on the stage at diagnosis [9]. A critical challenge in the clinical management of EOC is the emergence of drug resistance, which is the primary cause of treatment failure and disease recurrence [10]. Although many patients initially respond to first-line platinum-based chemotherapy, tumor cells frequently acquire resistance mechanisms that render subsequent treatments less effective [11]. This phenomenon is driven by a complex interplay of factors culminating in multidrug resistance [12]. Key mechanisms underlying drug resistance include impaired drug uptake (mediated by pathways such as PI3K/Akt/mTOR) [13], increased drug efflux (regulated by Wnt/β-catenin and Notch signaling) [14], enhanced DNA repair capacity (e.g., BRCA1/2 and PARP-dependent pathways) [15], dysregulation of apoptosis (via Akt and NF-κB signaling) [16], and overactivation of autophagy (through AMPK/mTOR and Beclin-1 pathways) [17].

Long noncoding RNAs (lncRNAs) have emerged as key regulators of cancer biology, influencing various processes, including cell proliferation, apoptosis, invasion, and metastasis [18]. More recently, studies have highlighted their pivotal roles in chemoresistance, where they orchestrate molecular pathways that enable cancer cells to evade therapeutic effects [19]. For instance, lncRNAs such as MALAT1 and HOTAIR have enhanced drug resistance by modulating epithelial–mesenchymal transition (EMT), autophagy, and DNA repair mechanisms [20, 21]. Additionally, NEAT1 has been implicated in promoting cisplatin resistance through its interaction with the Wnt/β-catenin pathway [22], while H19 contributes to resistance by regulating P-glycoprotein expression [23]. These findings illustrate the diversity of mechanisms by which lncRNAs facilitate resistance, highlighting their potential as biomarkers and therapeutic targets. Recent studies have also emphasized the tissue specificity and stability of lncRNAs, particularly in circulating serum and exosomal forms, making them attractive candidates for non-invasive biomarkers [24]. For example, circulating levels of HULC and UCA1 have been associated with resistance to chemotherapeutic agents in breast and bladder cancers, respectively, suggesting that similar strategies could be employed for EOC [25]. However, despite these advancements, research on lncRNA-mediated chemoresistance in EOC remains limited. Identifying and characterizing specific lncRNAs involved in EOC drug resistance could provide critical insights into disease progression and open avenues for personalized medicine.

This study aims to address this gap by investigating the expression profiles of seven specific lncRNAs—SNHG7, TUG1, XIST1, PRLB, TLR8-AS1, ZFAS1, and PVT1—in chemotherapy-resistant EOC cell lines and patient serum samples. These lncRNAs were identified through bioinformatics screening of public datasets and cross-referenced with prior studies on epithelial ovarian cancer and other malignancies. Through an experimental validation, the study evaluates their potential as non-invasive biomarkers and therapeutic targets in EOC.

## Materials and methods

Details of the experimental workflow are provided in Supplementary Table 1.

### Sample collection

Blood samples were collected from 25 histopathologically confirmed epithelial ovarian cancer patients and 23 healthy controls at the Department of Medical Oncology, Pamukkale University, from November 2021 to April 2023. The patient group provided samples before treatment and again six months post treatment. All participants provided written informed consent, and the study received ethical approval from Pamukkale University’s Faculty of Medicine Ethics Committee. The collected samples were stored at -80 °C for further analysis.

### Patient selection

Patients included in this study were selected based on stringent inclusion and exclusion criteria to ensure the relevance and validity of the findings. Inclusion criteria required a confirmed diagnosis of epithelial ovarian cancer via histopathological evaluation and the availability of detailed clinical and demographic data. Exclusion criteria eliminated individuals with concurrent malignancies, completion of first-line chemotherapy with carboplatin and paclitaxel, incomplete records, or non-standard treatments. The patient cohort, as detailed in Table 1, included diverse clinicopathological characteristics representative of EOC populations, such as tumor subtypes (e.g., high-grade serous carcinoma, low-grade serous carcinoma, and endometrioid carcinoma) and advanced-stage disease (84% in stages IIIA-IV). Additionally, hormonal receptor status (ER + /PR + in 84% of cases) and specific features like angiovascular invasion (20%) and unilateral or bilateral ovarian involvement were recorded to provide a comprehensive overview of the cohort. A control group of 23 healthy individuals matched by age and sex was included to ensure comparability.

**Table 1:** Detailed clinicopathological data of EOC patients

Clinicopathological features	Patient group (*n* = 25)
Average age	71.28
Smoking status	
Smoker	13 (52%)
Non-smoker	12 (48%)
Tumor type	
High-grade serous carcinoma	9 (36%)
Low-grade serous carcinoma	2 (8%)
Endometrioid carcinoma	2 (8%)
Malignant epithelial tumor metastasis	5 (20%)
Other (Serous Metastatic Carcinoma)	7 (28%)
Hormonal status	
ER + /PR +	21 (84%)
ER + /PR-	4 (16%)
ER-/PR-	0 (0%)
Histopathological condition	
Angiovascular Invasion (AVI)	
Present	5 (20%)
Absent	20 (80%)
Ovarian capsule condition	
Intact	19 (76%)
Ruptured	6 (24%)
Ovarian involvement	
Unilateral (Left / Right)	23 (92%)
Left	4 (16%)
Right	19 (76%)
Bilateral	2 (8%)

### Cell culture

This study utilized the drug-sensitive ovarian cancer cell lines OVCAR3 and SKOV3, which were provided by Pamukkale University’s Department of Medical Genetics. OVCAR3 cells were cultured in RPMI medium supplemented with 20% fetal bovine serum (FBS), while SKOV3 cells were maintained in McCoy’s 5A medium supplemented with 10% FBS. Both cell lines were subcultured every 5–7 days.

### Dose‒response assay

A dose‒response assay was conducted to assess the response of the OVCAR3 and SKOV3 cell lines to varying concentrations of carboplatin and paclitaxel. The cells were seeded in 96-well plates and treated with serial dilutions of the drugs after a 24-h incubation. Following 24 h of drug exposure, cell viability was measured via CVDK-8 (NutriCulture CVD Kit-8), and absorbance values were read at 450 nm to determine effective drug concentrations for further experiments. These values were directly correlated with cell viability, enabling the establishment of drug-resistant cell lines.

### Development of chemotherapy-resistant cell lines

Drug-resistant OVCAR3 and SKOV3 cell lines were established using an intermittent dosing protocol with carboplatin and paclitaxel. Initially, cells were treated with predetermined sublethal concentrations of either carboplatin or paclitaxel for a 4-day treatment phase, representing the minimum concentration tolerated by the cells. This was followed by a 3-day recovery period in a drug-free culture medium, allowing cells to adapt and regain proliferative capacity. This stepwise escalation strategy facilitated the progressive development of drug resistance while ensuring cell viability and stability [26].

### Cell viability assay

Cell viability following drug treatment was evaluated using the MTT assay and the CVDK-8 kit (Ecotec Biotechnology, UK). Parental and drug-resistant OVCAR3 and SKOV3 cell lines were seeded in 96-well plates at a density of 3,000 cells per well and incubated overnight to achieve 40–50% confluency. Cells were then treated with varying concentrations of carboplatin or paclitaxel. After 12, 24, and 48 h of drug exposure, 10 µL of CVDK-8 solution was added to each well, followed by an additional 4-h incubation. The absorbance was measured at 450 nm using a SpectraMax® iD3 Multi-Mode Microplate Reader (Molecular Devices, USA). The absorbance values were used to quantify cell viability relative to untreated controls.

### Isolation and quantitative real-time polymerase chain reaction (qRT‒PCR)

RNA was extracted from cell lines and serum samples via a standardized protocol (Bio‒Rad, USA). The isolated RNA was reverse transcribed into cDNA via the iScript™ cDNA Synthesis Kit (Bio-Rad, USA) following the manufacturer’s instructions. The cDNA was used to evaluate the expression levels of lncRNAs via qRT‒PCR on a Bio-Rad CFX96 Touch machine, employing Universal SYBR® Green Supermix for precise quantification. qRT-PCR was performed to analyze the expression levels of selected lncRNAs using SYBR Green chemistry. The housekeeping gene GAPDH was used as an internal control for normalization due to its stable expression across all experimental conditions [27]. Negative controls (no-template controls, NTCs) were included in each run to rule out contamination, and technical replicates (triplicates) were performed for each sample to ensure reproducibility. The reaction conditions were as follows: an initial denaturation step at 95 °C for 10 min, followed by 40 cycles of amplification comprising denaturation at 95 °C for 10 s, annealing at 63 °C for 15 s, and extension at 72 °C for 5 s. Melting curve analysis was performed by gradually increasing the temperature from 65 °C to 90 °C to confirm specificity. The sequences of primers used for each lncRNA are provided in Table 2. Relative lncRNA expression levels were calculated via the Pfaffl method (efficiency-corrected ΔΔCt) [28].

**Table 2:** RT‒qPCR primer sequences for targeted lncRNA analysis

LncRNA	Forward primer (5′–3′)	Reveres primer (3′–5′)
PVT1	TTCAGCACTCTGGACGGACTTG	GATGCAGCTCCTCAGATGAACC
PRLB	ACGCCATGTTGGGAGACTTC	TGAAAGCCCAGGGTCAACTC
SNHG7	CAGCCGCTTGTGTTCTTGATTC	AGTCCATCACAGGCGAAGTCAC
TUG1	TCACAACAGGAAGGACCATTGC	CGATAGATGAGGTTCCAGGTGC
TLR8-AS1	AGCTACTGTGCGTCCATGTT	ATCTCACCCGTGTCTTCCCT
XIST	GTTCTTAAAGCGCTGCAATTCG	AGAACCCCAAGTGCAGAGAGA
ZFAS1	GGAGGTTCAGGAAGCCATTCG	GCGTATGAAGCCTGACTGCAAC
GAPDH	ACAACTTTGGCATTGTGGAA	GATGCAGGGATGATGTTCTG

### Statistical analysis

The quantitative data are expressed as the means ± standard deviations (SDs) of the results from three independent experiments. Comparisons between two groups were performed via Student’s t test, with statistical significance set at a p value of < 0.05. When the assumptions for parametric testing were met, one-way ANOVA was employed to assess the mean differences across multiple independent groups.

## Results

### Data acquisition and analytical framework

A comprehensive bioinformatics workflow was employed to identify lncRNA candidates associated with chemoresistance in EOC. RNA sequencing data were obtained from two publicly available datasets: The Cancer Genome Atlas Ovarian Cancer Dataset (TCGA-OV) [29] and Gene Expression Omnibus (GEO) Dataset GSE165897 [30]. The TCGA-OV dataset includes RNA-seq data for 489 tumor samples, of which 309 were utilized for this analysis based on the availability of complete clinical and molecular annotations. The GEO dataset GSE165897 provides longitudinal single-cell RNA-seq data from 11 patients with metastatic ovarian cancer, covering both treatment-naïve and post-chemotherapy conditions. RNA-seq data were normalized and quality-checked using the DESeq2 pipeline. Chemo-resistant and chemo-sensitive samples were categorized based on clinical annotations in the respective datasets. Differentially expressed lncRNAs (DELs) were identified using DESeq2, applying thresholds of |log2 fold-change|> 1 and adjusted p value < 0.05 to select significant candidates. Co-expressed genes were identified via Pearson correlation analysis (r > 0.7), and functional enrichment analysis was conducted using the Database for Annotation, Visualization, and Integrated Discovery (DAVID) [31], focusing on KEGG pathways and GO biological processes to elucidate the roles of DELs in chemoresistance mechanisms. Interactions between identified lncRNAs and proteins were predicted using LncTAR [32], providing insights into the involvement of these lncRNAs in critical chemoresistance-related pathways.

**Fig. 1:**
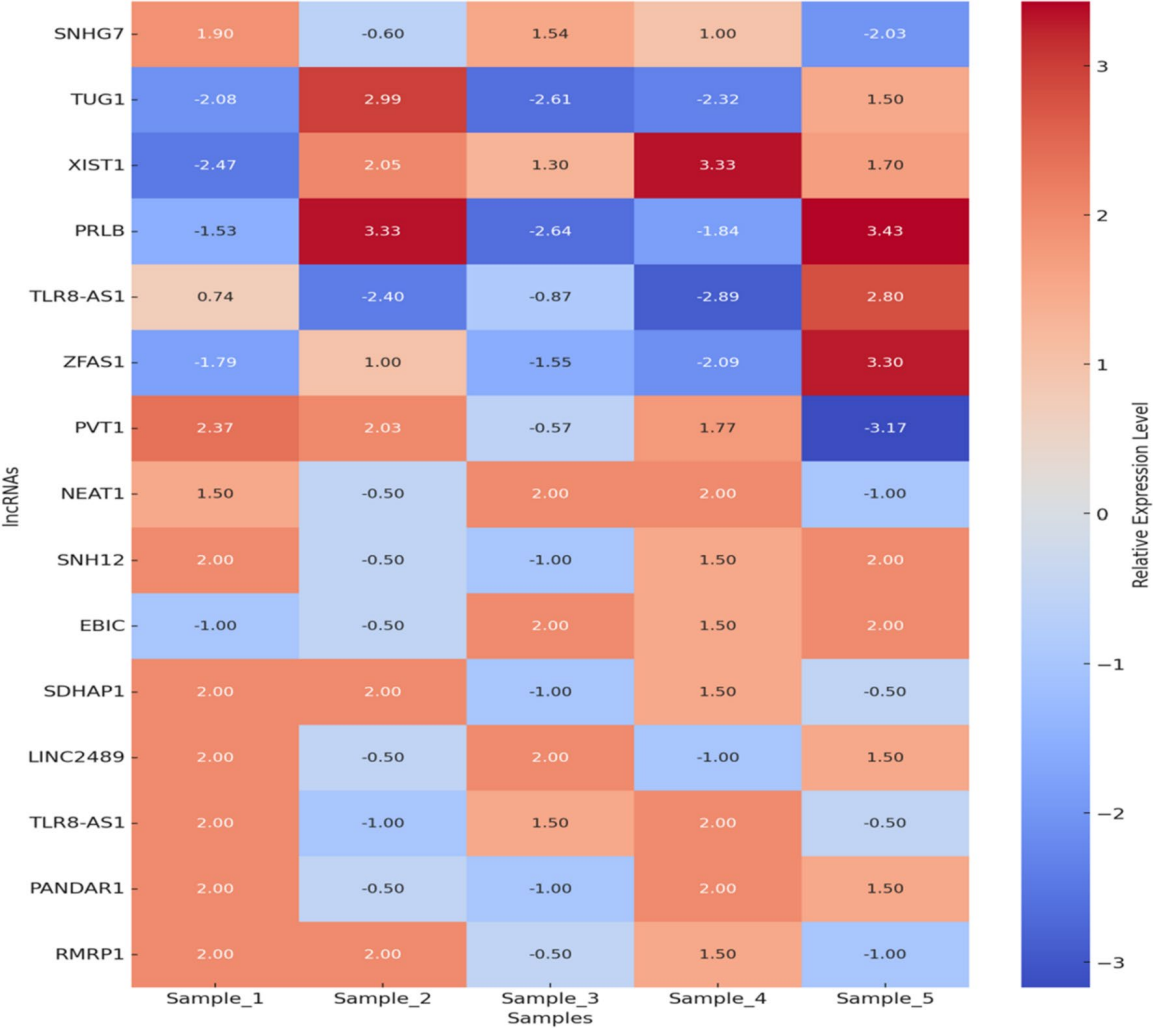
Heatmap of Differentially Expressed lncRNAs representing the expression levels of lncRNAs across 5 samples, with values ranging from downregulation (blue) to upregulation (red)

**Fig. 2:**
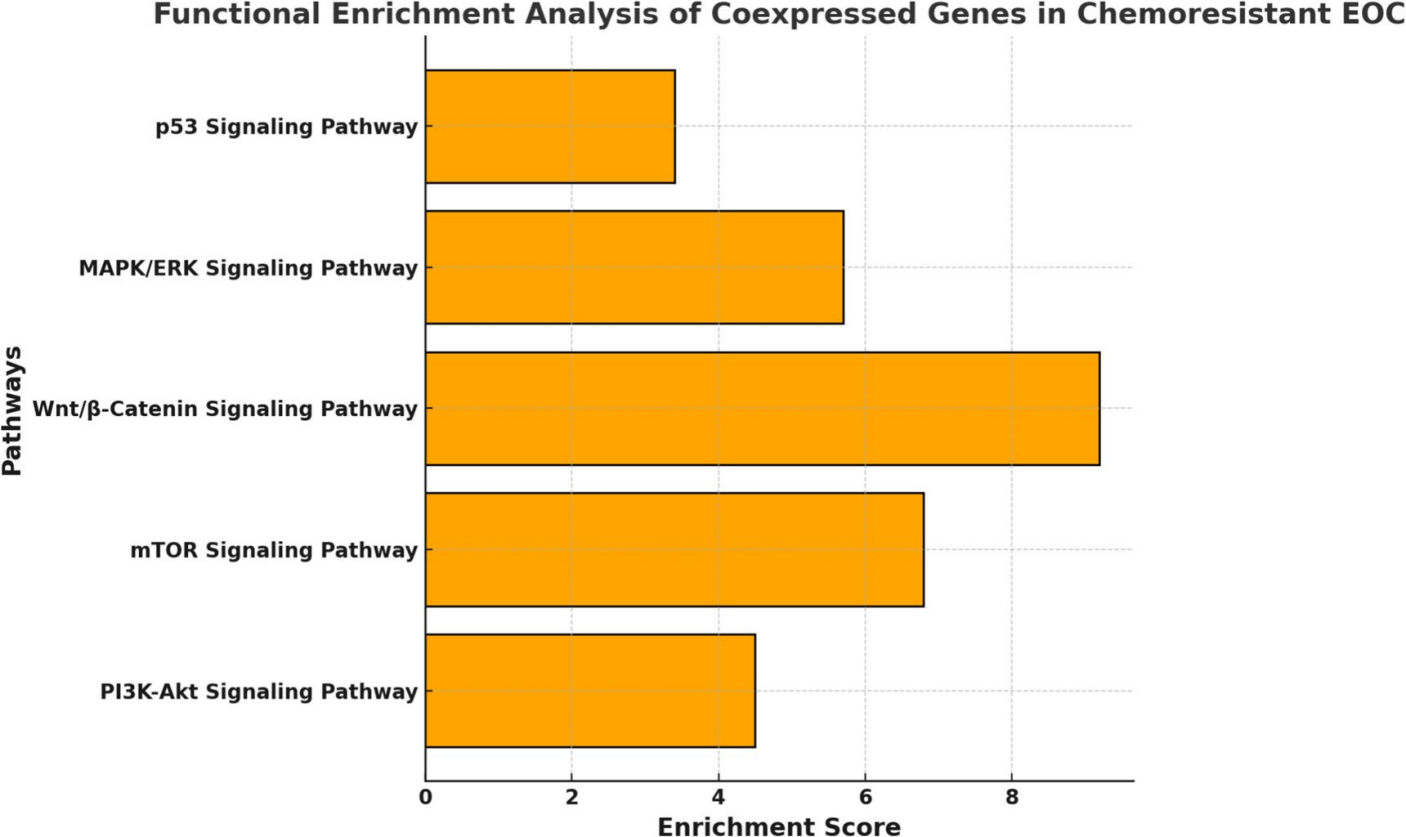
Functional enrichment analysis of coexpressed genes in chemo-resistant EOC. The bar chart highlights key signaling pathways, including PI3K-Akt, mTOR, Wnt/β-Catenin, MAPK/ERK, and p53, with their respective enrichment scores, indicating their potential involvement in lncRNA-mediated chemoresistance mechanisms

Results were visualized using heatmaps to display differentially expressed lncRNAs (Fig. 1), and bar charts for functional enrichment analyses (Fig. 2). This integrative approach enabled the identification of key lncRNAs contributing to chemoresistance and provided insights into their potential roles in EOC progression.

### Clinicodemographic comparison of epithelial ovarian cancer patients and controls

To assess the baseline characteristics of the study population, clinicodemographic data from 25 EOC patients and 23 healthy controls were compared (Table 3). The median age was 71.28 years for EOC patients and 69.5 years for controls, with no statistically significant difference between the groups (p = 0.523). Key lifestyle factors such as smoking and alcohol consumption were also comparable between groups. Smoking rates were 53% in patients and 52% in controls (p = 0.974), while alcohol consumption rates were 40% in both groups (p = 0.261). Similarly, the proportion of participants with a family history of cancer was 12% in the patient group and 8.7% in the control group, with no significant difference (p = 0.108). Additionally, there was no significant variation in the use of hormonal therapy (4% in both groups, p = 0.217) or the presence of benign ovarian conditions (12% in patients vs. 4.35% in controls, p = 0.347). These findings indicate that the patient and control groups were well matched in terms of clinicodemographic characteristics, ensuring homogeneity and minimizing potential confounding effects in subsequent analyses.

**Table 3:** Clinicodemographic characteristics of patients in the epithelial ovarian cancer patient group compared with those in the control group

		EOC Patient Group (*n* = 25)	Control Group (*n* = 23)	Between-Group p value
Age (median)		71.28	69.5	*p* = 0.523
Smoking	Yes	14 (53%)	12 (52%)	*p* = 0.974
	No	11 (44%)	11 (47.83%)	
Alcohol	Yes	10 (40%)	9 (40%)	*p* = 0.261
	No	15 (60%)	12 (60.87%)	
Family history of cancer	Yes	3 (12%)	2 (8.70%)	*p* = 0.108
	No	22 (88%)	21 (91.30%)	
Hormonal therapy use	Yes	1 (4%)	1 (4.35%)	*p* = 0.217
	No	24 (96%)	22 (95.65%)	
Benign ovarian conditions	Yes	3 (12%)	1 (4.35%)	*p* = 0.347
	No	22 (88%)	22 (95.65%)	

### Optimization of drug exposure for resistance development in OVCAR3 and SKOV3 cells

**Fig. 3:**
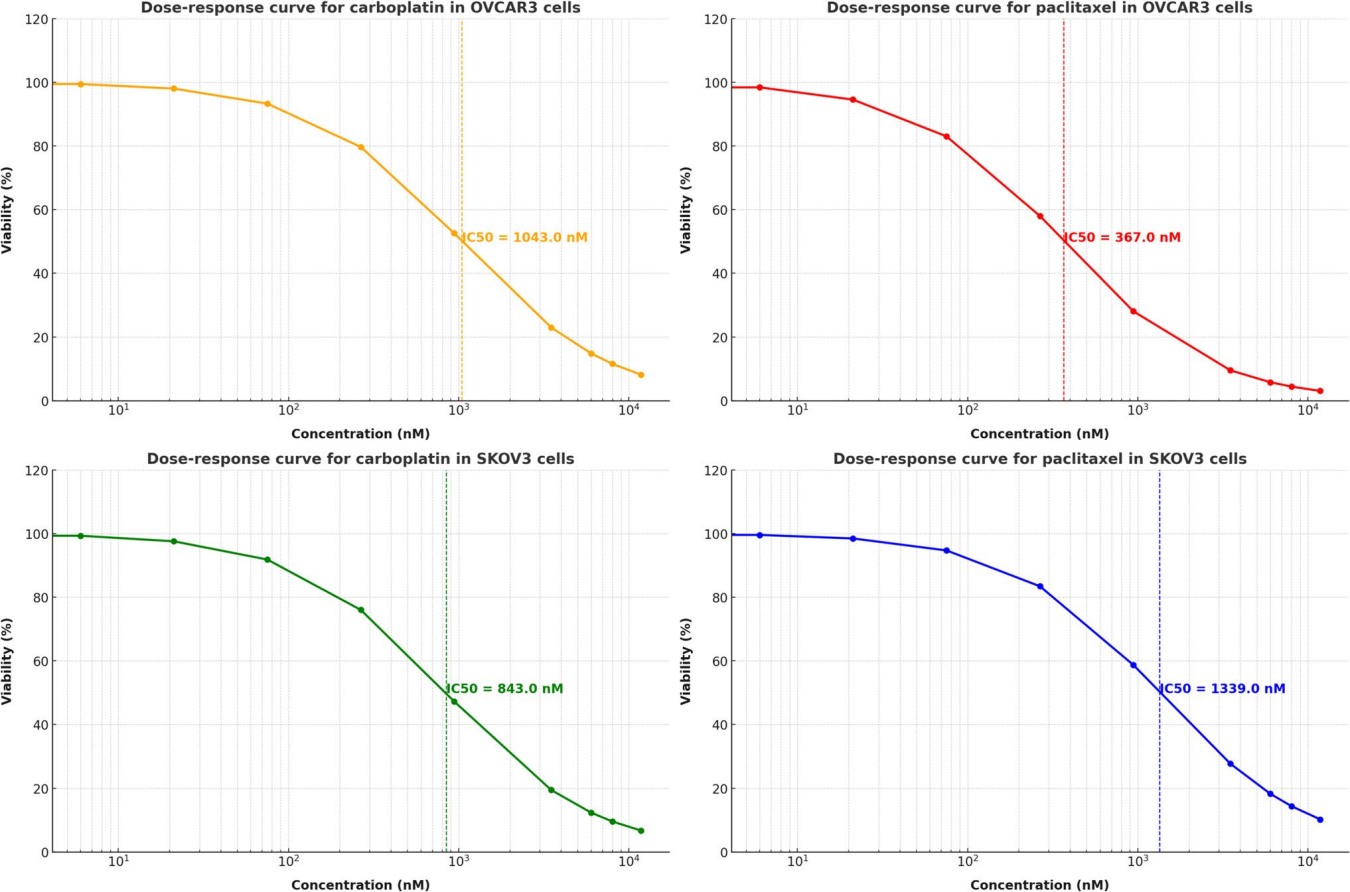
Sigmoidal dose–response curves illustrating the baseline sensitivity of OVCAR3 and SKOV3 cell lines to carboplatin and paclitaxel. The plots display cell viability (%) across varying drug concentrations (nM) at the start of the study, with IC₅₀ values indicated by dashed vertical lines. These IC₅₀ values represent the initial drug sensitivity of the cells, providing a reference for assessing subsequent changes in resistance over the course of treatment

To generate carboplatin- and paclitaxel-resistant OVCAR3 and SKOV3 cell lines, drug concentrations were gradually increased based on pharmacokinetic data [26]. Cells were continuously exposed to escalating doses of carboplatin (2.3–18.5 μg/ml for OVCAR3, 0.7–2 μg/ml for SKOV3) and paclitaxel (0.0023–0.1 µg/ml) over multiple passages. The optimal resistance-inducing concentration range was determined as 10–15 μg/ml for OVCAR3 and 1–1.5 μg/ml for SKOV3 for carboplatin. Paclitaxel-resistant cell lines were maintained in 6–11.8 µM paclitaxel, simulating clinically relevant exposure. Cells were passaged under continuous drug pressure for 6 weeks until resistant populations stabilized. Growth inhibition assays were performed at carboplatin (2.64–15.78 µM) and paclitaxel (6–11.8 µM) to confirm resistance induction. Cell viability and IC50 values were determined using MTT assays (detailed in Sect. “Cell viability assay”). The resistant cell populations were maintained in their respective drug concentrations for subsequent experiments (Fig. 3).

OVCAR3 and SKOV3 cells were seeded separately into two flasks at a density of 1 × 10⁶ cells per 75 cm^2^ of surface area. One flask, designated as the control group (OVCAR3-Par/SKOV3-Par), remained untreated, while the other was assigned to the treatment group. Cells in the treatment group were exposed to the optimized drug concentrations outlined in Table 4.

**Table 4:** Resistant cell names used in this study

OVCAR3-Par		
OVCAR3-R–C	OVCAR3-R-TAX	OVCAR3- R-COM
SCOV3-Par		
SCOV3-R–C	SCOV3-R-TAX	SCOV3-R-COM

*OVCAR3-Par* Parental OVCAR3 cell line, *OVCAR3-R–C* Carboplatin-resistant OVCAR3 cell line, *OVCAR3-R-TAX* Paclitaxel-resistant OVCAR3 cell line, *OVCAR3-R-COM* Combination-resistant OVCAR3 cell line, *SCOV3-Par* Parental SCOV3 cell line, *SCOV3-R–C* Carboplatin-resistant SCOV3 cell line, *SCOV3-R-TAX* Paclitaxel-resistant SCOV3 cell line, *SCOV3-R-COM* Combination-resistant SCOV3 cell line

### Confirmation of drug resistance in the OVCAR3-R/SKOV3-R cell lines

**Fig. 4:**
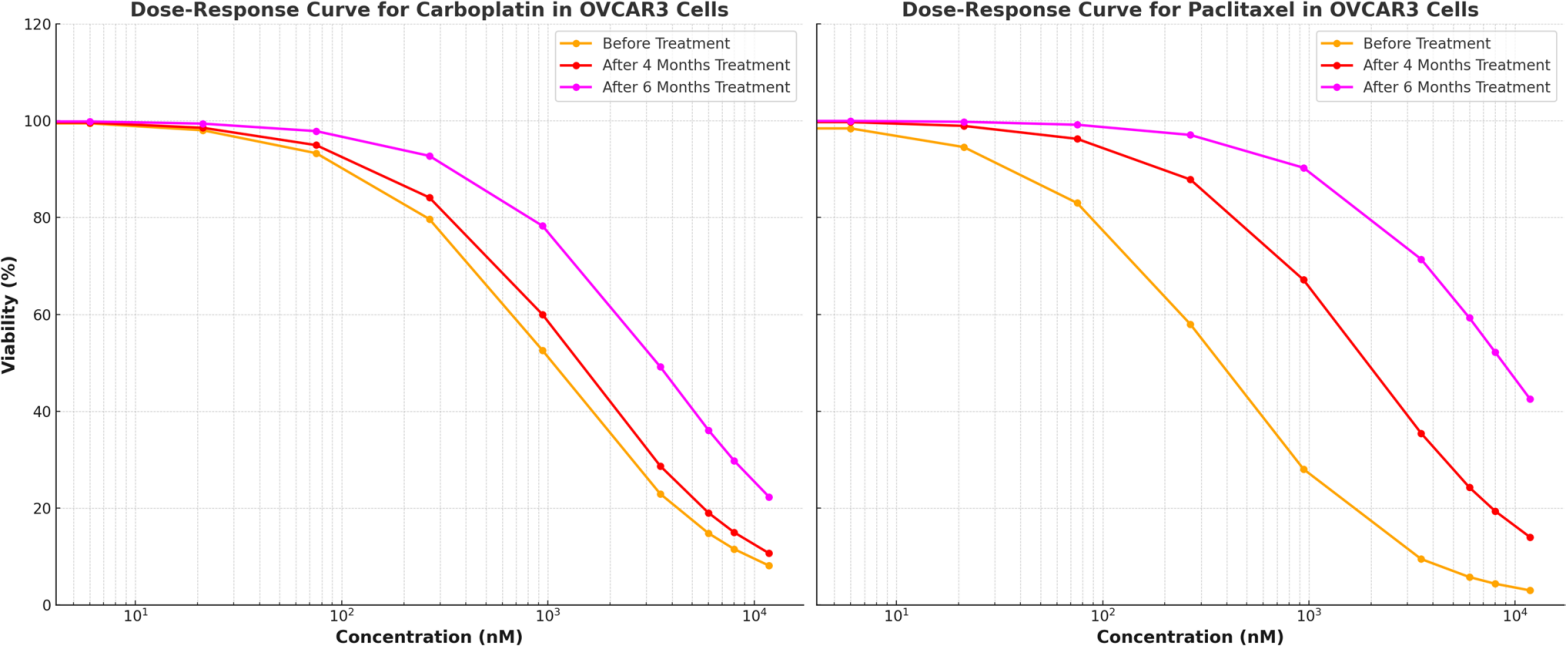
Sigmoidal dose–response curves for paclitaxel and carboplatin in OVCAR3 cells, illustrating cell viability (%) at varying drug concentrations (0 to 11,800 nM). The curves represent IC₅₀ values at different time points: before treatment (Carboplatin: 1043 nM, Paclitaxel: 367 nM), after 4 months of treatment (Carboplatin: 1410 nM, Paclitaxel: 1923 nM), and after 6 months of treatment (Carboplatin: 3390 nM, Paclitaxel: 8735 nM). The observed rightward shift in the dose–response curves over time reflects an increase in drug resistance. The x-axis is displayed logarithmically to emphasize trends across a broad concentration range

**Fig. 5:**
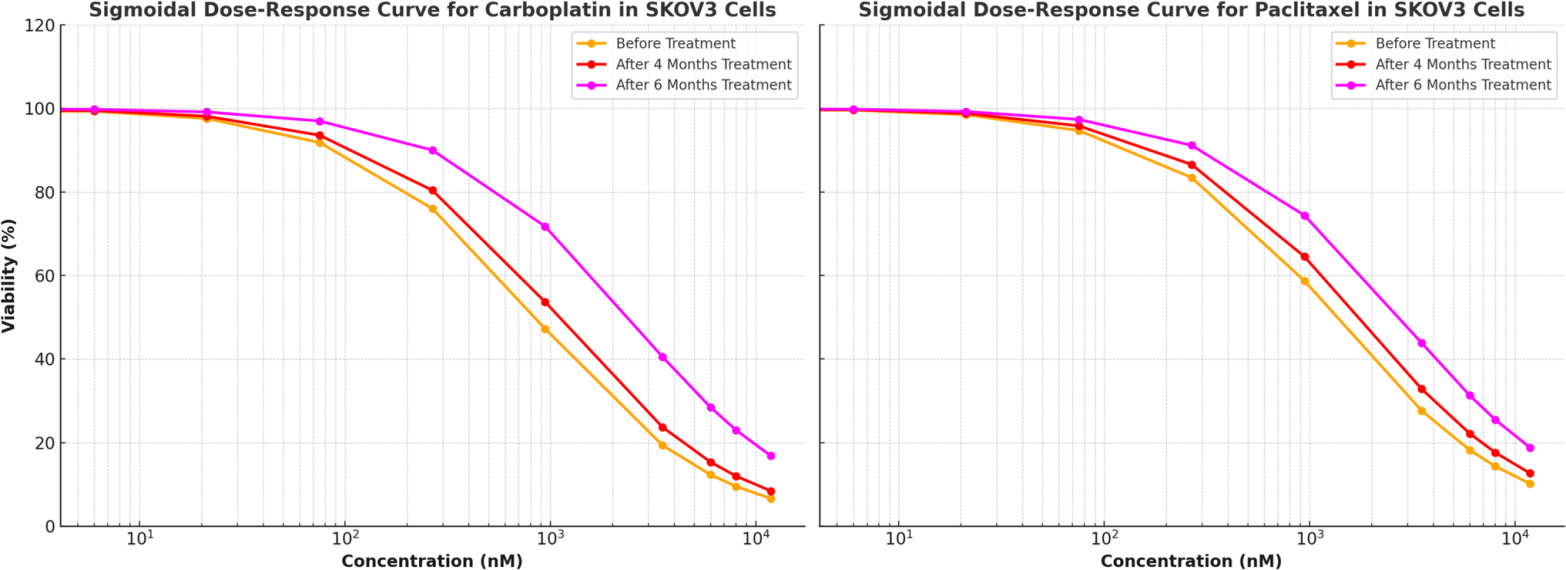
Sigmoidal dose–response curves for carboplatin and paclitaxel in SKOV3 cells, illustrating cell viability (%) at varying drug concentrations (0 to 11,800 nM). The curves represent IC₅₀ values at different time points: before treatment (Carboplatin: 843 nM, Paclitaxel: 1339 nM), after 4 months of treatment (Carboplatin: 1090 nM, Paclitaxel: 1712 nM), and after 6 months of treatment (Carboplatin: 2390 nM, Paclitaxel: 2735 nM). The observed rightward shift in the dose–response curves over time indicates increased drug resistance. The x-axis is displayed logarithmically to emphasize trends across the full range of concentrations

After six months of treatment, the sensitivity of OVCAR3 and SKOV3 cells to carboplatin and paclitaxel was re-evaluated. While the IC₅₀ values remained unchanged in OVCAR3-Par and SKOV3-Par cells, a significant increase was observed in OVCAR3-R and SKOV3-R cells, confirming the acquisition of drug resistance (Fig. 4,5). In carboplatin-treated OVCAR3 cells, the initial IC₅₀ value of 1.043 ± 0.03 µM increased to 3.391 ± 0.03 µM after six months, reflecting a 3.258-fold increase in resistance. Paclitaxel resistance was even more pronounced, with IC₅₀ values rising from 0.367 ± 0.02 µM to 8.735 µM, indicating a 23.764-fold increase in resistance. A similar pattern was observed in SKOV3 cells. The IC₅₀ for carboplatin increased from 0.843 ± 0.05 µM to 2.399 ± 0.02 µM over six months, corresponding to a 2.832-fold increase in resistance. Although this increase was less pronounced compared to OVCAR3 cells, SKOV3 cells still exhibited a significant rise in carboplatin resistance over time. These findings confirm the successful establishment of chemo-resistant cell lines.

**Fig. 6:**
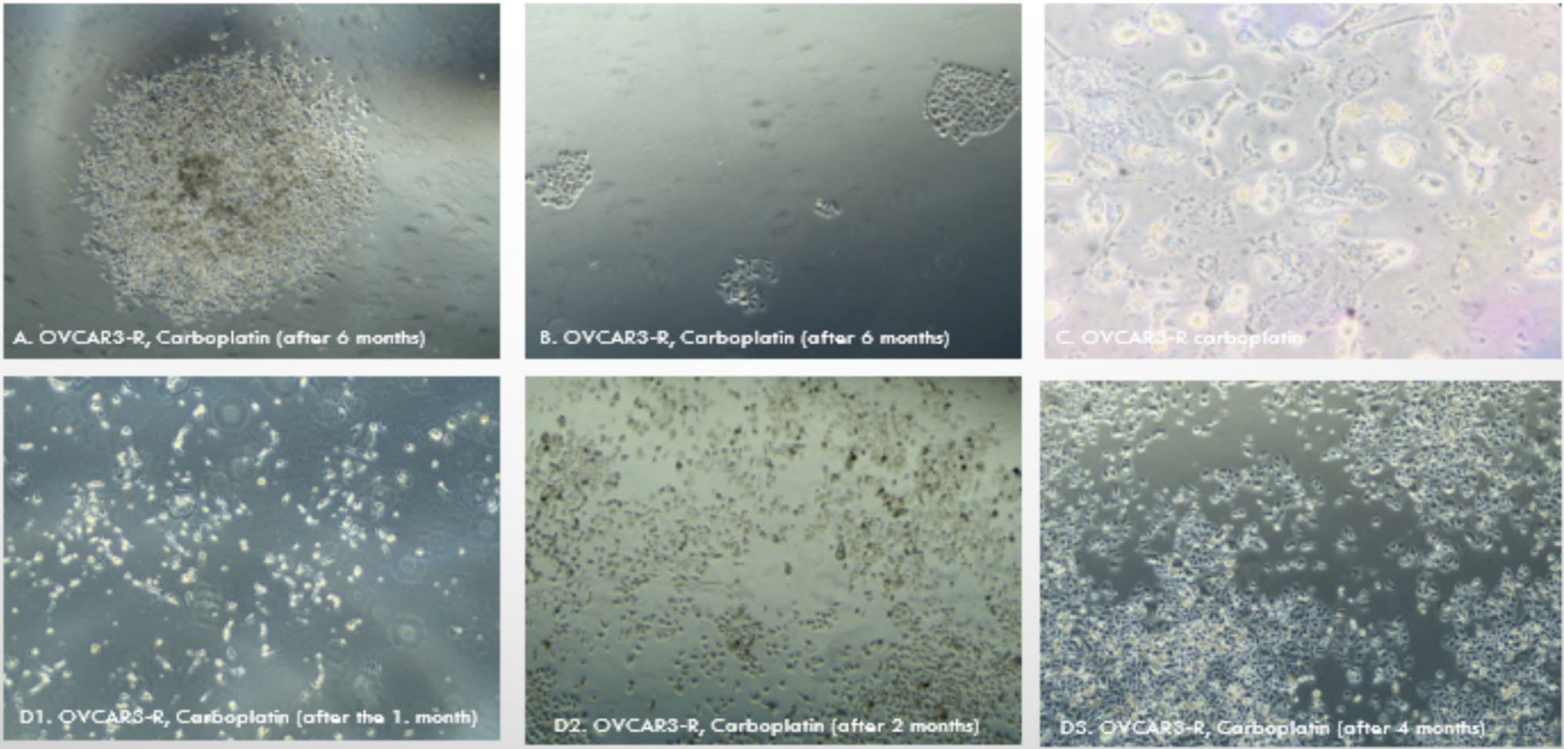
Morphological Adaptations in OVCAR3-R Cells During Carboplatin Resistance Development Observed via Phase-Contrast Microscopy. **A** After 6 months of carboplatin treatment, OVCAR3-R cells exhibited well-defined colony boundaries, indicating adaptation to drug exposure and the acquisition of resistance (PCM, 20 ×). **B** A loss of colony synapses was observed in OVCAR3-R cells after 6 months of treatment, suggesting a transition to a more invasive phenotype (PCM, 20 ×). **C** Resistant OVCAR3-R cells displayed an increase in cell size and volume, reflecting potential metabolic alterations associated with prolonged drug exposure (PCM, 40 ×). **D1**, **D2**, **D3** Progressive changes in cellular morphology and density were documented at different time points (1, 2, and 4 months) during resistance induction, demonstrating an incremental shift in cell organization and drug tolerance (PCM, 10 ×)

Throughout the resistance induction process, careful monitoring of cell morphology, viability, and growth kinetics was essential to ensure the development of persistent resistance rather than transient drug tolerance [25]. Notably, drug-resistant OVCAR3 and SKOV3 cells exhibited distinct colony boundaries, compact spheroid structures, and increased cell volume and spreading compared with their parental counterparts (Fig. 6). These morphological changes suggest an epithelial–mesenchymal transition (EMT)-like process, which may be associated with enhanced cell–cell and extracellular matrix interactions [26, 33].

### Comparison of LncRNA expression profiles in various treatment conditions via RT-qPCR

**Fig. 7:**
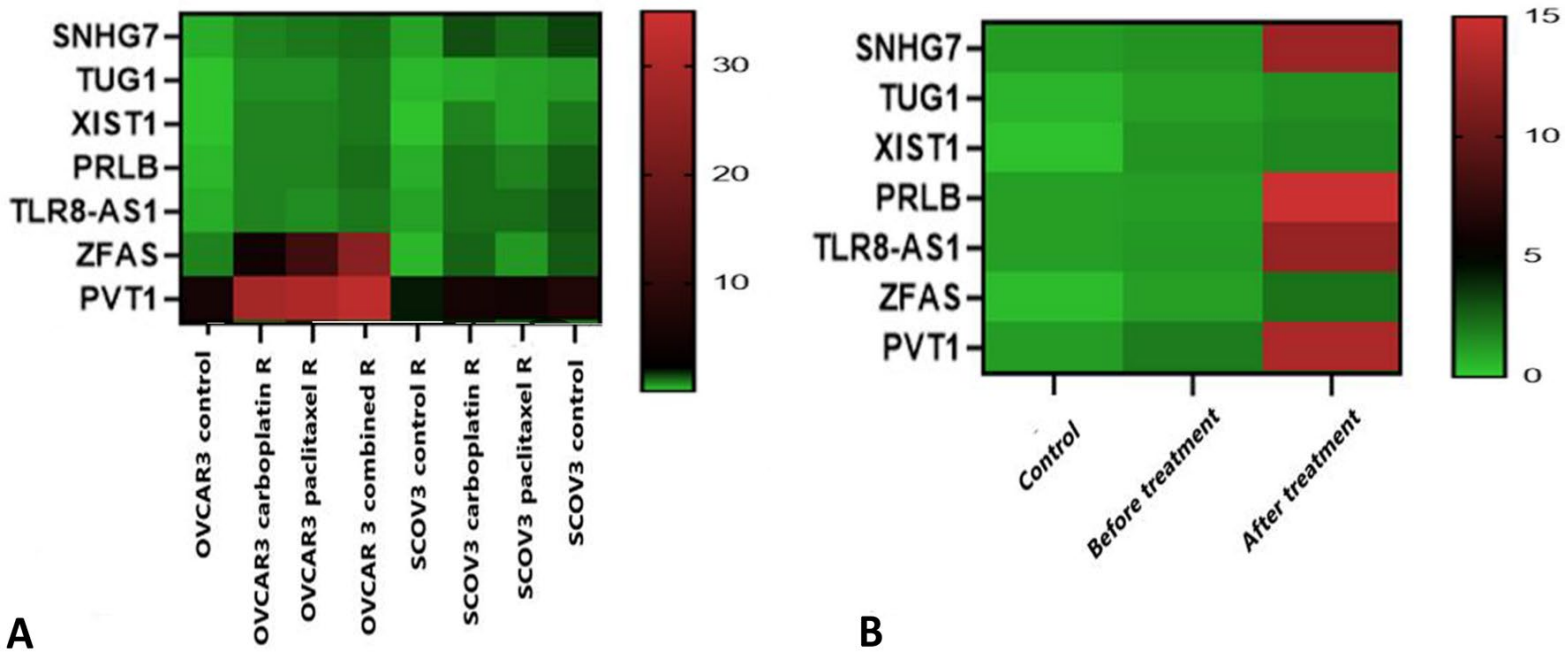
Expression profiles of various lncRNAs across different cancer cell lines and treatment conditions. Heatmap A illustrates the differential expression levels of various lncRNAs in ovarian cancer cell lines (OVCAR3 and SKOV3), comparing carboplatin- and paclitaxel-resistant cells (R) to those subjected to combined treatment. Heatmap B depicts the changes in lncRNA expression in the control groups, before and after treatment samples. (Upregulation of lncRNAs is represented by red, whereas downregulation is indicated by green) (Color figure online)

This study assessed the expression profiles of seven lncRNAs previously implicated in chemoresistance in EOC. Reverse transcription quantitative PCR (RT-qPCR) was conducted on carboplatin- and paclitaxel-resistant OVCAR3-R and SKOV3-R cell lines, revealing notable differences in expression compared with their parental counterparts (Fig. 7). Among the analyzed lncRNAs, SNHG7, TUG1, XIST1, PRLB, TLR8-AS1, ZFAS1, and PVT1 exhibited significantly higher expression levels in resistant cells (P < 0.05). Additionally, RT-qPCR analysis of serum samples from 25 EOC patients before and after chemotherapy, as well as 23 healthy controls, revealed significant differential expression patterns. One-way ANOVA indicated statistically significant differences between groups, with fold changes presented in Table 5.

**Table 5:** Summary of lncRNA expression levels in control, pre- and post-chemotherapy serum samples, and drug-resistant OVCAR3/SCOV3 cell lines

Sample type	SNHG7	TUG1	XIST	PRLB	TLR8-AS1	ZFAS1	PVT1
Control group	2.00 ± 0.06	3.3 ± 0.04	4.2 ± 0.01	11.4 ± 0.02	11.7 ± 0.02	36.8 ± 0.02	25.4 ± 0.06
Pre-chemotherapy	13.0 ± 0.01	30.3 ± 0.04	13.2 ± 0.01	30.11 ± 0.01	40.2 ± 0.01	40.01 ± 0.07	92.4 ± 0.03
Post-chemotherapy	69.9 ± 0.03	155.6 ± 0.04	105.9 ± 0.02	155.6 ± 0.01	167.3 ± 0.01	360.5 ± 0.04	463.4 ± 0.05
OVCAR3-parental	0.4 ± 0.05	0.2 ± 0.05	0.2 ± 0.01	0.3 ± 0.13	0.4 ± 0.03	0.8 ± 0.07	5.4 ± 0.03
OVCAR3-R–C	0.8 ± 0.01	0.7 ± 0.04	0.8 ± 0.01	0.8 ± 0.01	0.8 ± 0.01	15.1 ± 0.04	28.5 ± 0.05
OVCAR3-R-PTX	0.9 ± 0.02	0.7 ± 0.03	0.7 ± 0.01	0.8 ± 0.03	0.7 ± 0.01	12.1 ± 0.03	29.7 ± 0.02
OVCAR3-R-COM	1.0 ± 0.03	0.9 ± 0.01	0.9 ± 0.03	1.0 ± 0.01	0.9 ± 0.01	24.0 ± 0.02	32.3 ± 0.13
SCOV3-Parental	0.5 ± 0.05	0.3 ± 0.07	0.2 ± 0.01	0.4 ± 0.05	0.5 ± 0.03	0.3 ± 0.05	1.8 ± 0.03
SCOV3-R–C	1.3 ± 0.09	0.4 ± 0.09	0.8 ± 0.02	1.0 ± 0.15	1.0 ± 0.02	1.1 ± 0.04	5.5 ± 0.07
SCOV3-R-PTX	1.0 ± 0.08	0.5 ± 0.01	0.5 ± 0.01	0.8 ± 0.10	1.0 ± 0.01	0.6 ± 0.09	4.9 ± 0.03
SCOV3-R-COM	1.4 ± 0.04	0.6 ± 0.03	0.9 ± 0.01	1.2 ± 0.08	1.3 ± 0.01	1.2 ± 0.07	7.2 ± 0.10

Data are presented as mean ± standard deviation (SD). **b** Expression levels were normalized to GAPDH and calculated using the Pfaffl method. *p < 0.05 indicates statistically significant differences between groups

## Discussion

**Fig. 8:**
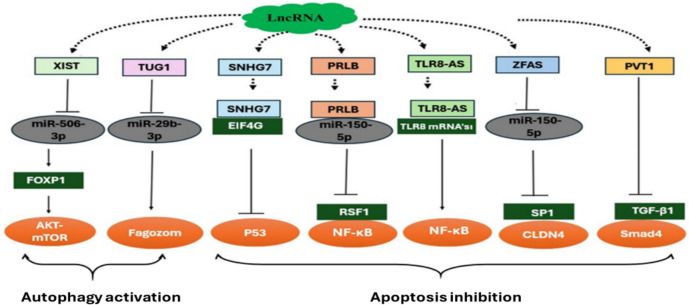
Mechanisms of action of lncRNAs with significant expression. This figure illustrates how the identified lncRNAs influence various cellular pathways, highlighting their roles in key processes such as apoptosis, autophagy, and chemoresistance

In epithelial ovarian cancer, chemoresistance poses a significant barrier to successful treatment, ultimately leading to poor patient outcomes. Long noncoding RNAs play pivotal roles in driving resistance by orchestrating critical molecular processes, such as apoptosis inhibition, autophagy activation, and drug efflux regulation [11, 34]. This study investigated the expression profiles and molecular roles of seven lncRNAs in a Turkish cohort of EOC patients both before and after chemotherapy. Specifically, the study aimed to evaluate their potential as non-invasive biomarkers for early detection, treatment monitoring, and personalized therapy. Using a combination of in silico analysis, including a TCGA transcriptome survey, and experimental validation with EOC cell lines (OVCAR3 and SKOV3), we identified significant dysregulation of the lncRNAs SNHG7, TUG1, XIST1, PRLB, TLR8-AS1, ZFAS1, and PVT1 in chemotherapy-resistant versus sensitive cell lines, as well as in serum samples from patients and healthy controls. These lncRNAs were selected based on their involvement in key cellular processes, including apoptosis and autophagy, which are directly linked to chemotherapy resistance. Our results revealed a marked upregulation of these lncRNAs in post-chemotherapy serum samples compared with pre-chemotherapy and healthy control samples, particularly in patients exhibiting chemoresistance. According to their molecular mechanisms, these lncRNAs were categorized into two major groups: those involved in apoptosis dysregulation and those associated with autophagy (Fig. 8).

The results of this study suggest a potential role for SNHG7 in chemotherapy resistance, as its expression was significantly elevated in resistant OVCAR3 and SKOV3 cell lines and post-chemotherapy serum samples. Its interaction with EIF4G2 and p53 may contribute to enhanced survival, migration, and invasion, while reducing apoptosis [35, 36]. However, further research is needed to determine whether SNHG7’s role in resistance is a direct regulatory mechanism or part of a broader cellular adaptation to chemotherapy.

Similarly, ZFAS1 was found to be upregulated in drug-resistant cell lines and patient serum samples, aligning with the previous reports linking its overexpression to increased resistance to paclitaxel and carboplatin in EOC [37]. While these findings indicate an association with drug resistance pathways, functional studies are necessary to determine whether ZFAS1 directly modulates chemoresistance or acts through secondary regulatory mechanisms.

TLR8-AS1 expression was also elevated after chemotherapy, suggesting a possible involvement in immune evasion and apoptosis inhibition. Its previously reported interaction with NF-κB and PI3K/AKT signaling pathways supports its potential role in chemotherapy resistance [38]. However, the precise mechanisms by which TLR8-AS1 contributes to chemoresistance in EOC remain unclear, requiring further investigation.

PRLB was significantly overexpressed in chemotherapy-resistant cell lines and post-chemotherapy serum samples, suggesting a link to chemoresistance. Its proposed mechanism involves downregulation of miR-150-5p, leading to RSF1 protein upregulation and activation of NF-κB signaling, which has been implicated in apoptosis dysregulation and drug resistance [39]. These findings highlight PRLB as a potential biomarker, but additional studies are required to establish its functional significance.

PVT1 was notably upregulated in resistant cell lines and patient serum samples, supporting its potential role in ovarian cancer progression and treatment resistance. Previous studies suggest that PVT1 inhibition reduces cisplatin resistance by blocking the JAK2/STAT3/PD-L1 signaling pathway [40]. While these results highlight its clinical relevance, further validation in larger patient cohorts is necessary.

TUG1 and XIST1 were also linked to chemoresistance and may serve as prognostic markers for disease recurrence and treatment response. TUG1 overexpression correlated with increased autophagy and reduced apoptosis, promoting survival under cytotoxic stress [41]. Similarly, XIST1 was upregulated in resistant cells and post-chemotherapy serum samples, with studies suggesting that it enhances carboplatin resistance via the miR-506-3p/FOXP1 axis [42]. While these findings are promising, additional research is needed to confirm their mechanistic roles in drug resistance and EOC progression.

The identification of these serum-based lncRNAs as non-invasive biomarkers introduces a potential strategy for monitoring disease progression and treatment response. Their application in personalized medicine could improve EOC management by enabling real-time tracking of molecular changes. However, larger validation studies are needed to determine the reliability of these lncRNAs as clinical biomarkers and their role in predicting treatment outcomes.

Despite these findings, several limitations must be considered. The relatively small cohort of Turkish EOC patients and cell lines may not fully represent EOC heterogeneity across different populations. Factors such as age, tumor stage, genetic mutations, and chemotherapy regimens—including variations in drug dosage and scheduling—could introduce variability in lncRNA expression patterns. Expanding future studies to multi-center cohorts with diverse ethnic and geographic representation could improve the generalizability of findings. Additionally, while this study identified significant lncRNA expression changes, functional validation through knockdown or overexpression experiments was not conducted. Subtle differences in sample handling and qRT-PCR procedures may also contribute to variability. Future research should integrate CRISPR/Cas9-mediated gene editing, RNA interference (RNAi), and advanced transcriptomic/proteomic analyses to establish causal relationships between lncRNAs and chemoresistance. Addressing these methodological limitations will be essential for translating these findings into clinical applications and improving therapeutic strategies for chemo-resistant EOC patients.

## Conclusion

This study establishes a framework for leveraging specific long noncoding RNAs in the non-invasive detection and management of chemoresistance in epithelial ovarian cancer. By categorizing seven lncRNAs into apoptosis-related (**SNHG7, ZFAS1, TLR8-AS1, PRLB, and PVT1**) and autophagy-related (**TUG1, XIST1**) groups, the findings suggest their dual potential as biomarkers and therapeutic targets. The significant upregulation of these lncRNAs in chemotherapy-resistant cell lines and post-chemotherapy serum samples underscore their probable association with blocking apoptosis and promoting autophagy to drive chemoresistance.

## Supplementary Information

Below is the link to the electronic supplementary material.

Supplementary file1 (DOCX 17 kb)

## Data Availability

The datasets generated and analyzed during the current study are available from the corresponding author on reasonable request.

## Abbreviations

EOC: Epithelial ovarian cancer
lncRNA: Long noncoding RNA
RT-qPCR: Reverse transcription quantitative polymerase chain reaction
TCGA: The cancer genome atlas
CRISPR: Clustered regularly interspaced short palindromic repeats
RNAi: RNA interference
PI3K: Phosphoinositide 3-kinase
AKT: Protein kinase B
mTOR: Mammalian target of rapamycin
NF-κB: Nuclear factor kappa B
JAK2: Janus kinase 2
STAT3: Signal transducer and activator of transcription 3
PD-L1: Programmed death-ligand 1
FOXP1: Forkhead box P1
EMT: Epithelial–mesenchymal transition
PVT1: Plasmacytoma variant translocation 1
HOTAIR: HOX transcript antisense RNA
TUG1: Taurine upregulated Gene 1
XIST: X-Inactive-specific transcript
ZFAS1: Zinc finger antisense 1
PRLB: Placental RNA-like beta
TLR8-AS1: Toll-like receptor 8 antisense RNA 1
OVCAR3: Human ovarian carcinoma cell line
SCOV3: Human ovarian serous carcinoma cell line
OVCAR3-Par: Parental OVCAR3 cell line
OVCAR3-R–C: Carboplatin-resistant OVCAR3 cell line
OVCAR3-R-TAX: Paclitaxel-resistant OVCAR3 cell line
OVCAR3-R-COM: Combination-resistant OVCAR3 cell line (Carboplatin + Paclitaxel)
SCOV3-R–C: Carboplatin-resistant SCOV3 cell line
SCOV3-R-TAX: Paclitaxel-resistant SCOV3 cell line
SCOV3-R-COM: Combination-resistant SCOV3 cell line (Carboplatin + Paclitaxel)
ANOVA: Analysis of variance
IC50: Half-maximal inhibitory concentration
