# Supplementary material for: Characterization of lncRNAs contributing to drug resistance in epithelial ovarian cancer
Source: Med Oncol. 2025 Feb 24;42(4):84. doi: 10.1007/s12032-025-02628-1 (PMC11850566; doi:10.1007/s12032-025-02628-1)
Supplement: Supplementary file 1 — Supplementary file1 (DOCX 17 kb) [file 12032_2025_2628_MOESM1_ESM.docx]

**Supplementary Table**

**Supplementary Table 1**: Experimental Workflow Overview

| \| **Step** \| \| --- \| | **Description** |
| --- | --- | --- |
| **Patient & Sample Collection** | Blood samples collected from 25 EOC patients and 23 healthy controls before and after chemotherapy. |
| **Cell Line Selection** | Drug-sensitive and drug-resistant OVCAR3 and SKOV3 cell lines were utilized for experimental validation. |
| **Bioinformatics Analysis** | lncRNA candidates were identified via TCGA and GEO databases, selecting key lncRNAs associated with EOC chemoresistance. |
| **Differential Expression Analysis** | qRT-PCR was performed on serum samples and cell lines to assess the expression profiles of SNHG7, TUG1, XIST1, PRLB, TLR8-AS1, ZFAS1, and PVT1. |
| **Cell Viability Assays** | Drug resistance was evaluated using MTT assays to determine IC50 values for paclitaxel and carboplatin before and after chemotherapy. |
| **Bioinformatics-Based Categorization of lncRNAs** | Identified lncRNAs were categorized into apoptosis-related and autophagy-related groups based on their predicted molecular associations, as suggested by existing literature and bioinformatics analyses. |
| **Correlation with Chemoresistance** | Expression changes were analyzed in relation to drug resistance mechanisms and treatment response. |
| **Conclusion & Future Directions** | Findings suggest these lncRNAs as potential noninvasive biomarkers, but further clinical validation is required for integration into personalized therapy. |
